# Tailorable Nanoporous Hydroxyapatite Scaffolds for Electrothermal Catalysis

**DOI:** 10.1021/acsanm.2c01915

**Published:** 2022-05-20

**Authors:** Jordi Sans, Marc Arnau, Joan Josep Roa, Pau Turon, Carlos Alemán

**Affiliations:** † Departament d’Enginyeria Química, EEBE, Universitat Politècnica de Catalunya, C/Eduard Maristany, 10-14, Ed. I2, 08019 Barcelona, Spain; ‡ Barcelona Research Center in Multiscale Science and Engineering, Universitat Politècnica de Catalunya, C/Eduard Maristany, 10-14, 08019 Barcelona, Spain; § CIEFMA-Departament de Ciència i Eng. de Materials, Universitat Politècnica de Catalunya, Eduard Maristany 10-14, Ed. I, 08019 Barcelona, Spain; ∥ B. Braun Surgical, S.A.U. Carretera de Terrassa 121 Rubí, 08191 Barcelona, Spain; ⊥ Institute for Bioengineering of Catalonia (IBEC), The Barcelona Institute of Science and Technology, Baldiri Reixac 10-12, 08028 Barcelona, Spain

**Keywords:** amino acids, ammonium production, carbon fixation, decarbonization, ditrogen fixation, ethanol production, pluronic hydrogel, polarized hydroxyapatite

## Abstract

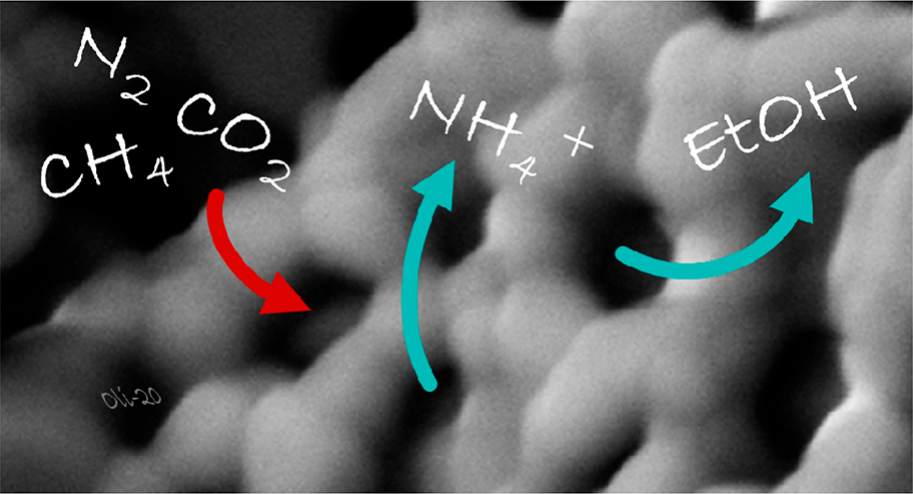

Polarized hydroxyapatite (HAp) scaffolds with customized architecture at the nanoscale have been presented as a green alternative to conventional catalysts used for carbon and dinitrogen fixation. HAp printable inks with controlled nanoporosity and rheological properties have been successfully achieved by incorporating Pluronic hydrogel. Nanoporous scaffolds with good mechanical properties, as demonstrated by means of the nanoindentation technique, have been obtained by a sintering treatment and the posterior thermally induced polarization process. Their catalytic activity has been evaluated by considering three different key reactions (all in the presence of liquid water): (1) the synthesis of amino acids from gas mixtures of N_2_, CO_2_, and CH_4_; (2) the production of ethanol from gas mixtures of CO_2_ and CH_4_; and (3) the synthesis of ammonia from N_2_ gas. Comparison of the yields obtained by using nanoporous and nonporous (conventional) polarized HAp catalysts shows that both the nanoporosity and water absorption capacity of the former represent a drawback when the catalytic reaction requires auxiliary coating layers, as for example for the production of amino acids. This is because the surface nanopores achieved by incorporating Pluronic hydrogel are completely hindered by such auxiliary coating layers. On the contrary, the catalytic activity improves drastically for reactions in which the HAp-based scaffolds with enhanced nanoporosity are used as catalysts. More specifically, the carbon fixation from CO_2_ and CH_4_ to yield ethanol improves by more than 3000% when compared with nonporous HAp catalyst. Similarly, the synthesis of ammonia by dinitrogen fixation increases by more than 2000%. Therefore, HAp catalysts based on nanoporous scaffolds exhibit an extraordinary potential for scalability and industrial utilization for many chemical reactions, enabling a feasible green chemistry alternative to catalysts based on heavy metals.

## Introduction

Mimicking the reactor morphologies observed in nature has attracted scientists for their drastically enhanced catalytic activity. Confining the volume of chemical reactions to the micro- and nanoscales offers numerous advantages, such as the increase of the surface-to-volume ratio and the control on the heat and mass transfer, which are translated to an enhanced final selectivity and efficiency.^1−3^ Indeed, development of nanoreactors and catalysts in the form of hollow or porous structures is currently a topic of great interest.^4−7^

In recent studies we reported the utilization of hydroxyapatite (HAp), Ca_10_(PO_4_)_6_(OH)_2_, for nitrogen and carbon fixation conversion in added valued molecules under mild reaction conditions,^8,9^ which postulates as a green and cheap alternative to conventional catalysts, showing an excellent selectivity behavior.^10^ Catalytic activation of HAp was achieved by applying a thermally stimulated polarization (TSP) treatment to sintered pellets previously obtained by compressing HAp powder. Although the TSP treatment causes the permanent alignment of the OH^–^ groups contained in the lattice through a specific direction and confers both electrical and electrochemical properties,^11,12^ the poorly porous structure of catalytic HAp (hereafter denoted HAp/c) pellets restricts the efficiency of the catalyst and, therefore, the final yield of the reactions.

The utilization of porous HAp/c inspired in HAp scaffolds recently developed for tissue engineering^13−17^ and air filtration applications^18^ represents an attractive approach for designing three-dimensional (3D) catalysts with greater specific surface area. Among other techniques, such as gas foaming,^19^ freeze-drying,^20^ and emulsification,^21^ 3D printing technology has recently gained importance due to its capacity for modeling the architecture and controlling the porosity, pore size, pore shape, and dimensions of the samples, allowing to differentiate between macropores and micropores (>50 μm and <10 μm, respectively).^22−24^ Within this context, 3D printing by means of direct-ink writing (i.e., robotic material extrusion) has been widely explored for biological applications due to the possibility of creating customized printable HAp inks through the addition of appropriate biocompatible polymers, depending on the requirements of each precise application.^25−28^ However, the choice of the suitable polymers might be a challenging task. While synthetic polymers, such as poly(lactic acid) (PLA)^27^ and polycaprolactone (PCL),^28^ present outstanding rheological properties, their hydrophobicity and processability at the high temperatures required for extrusion could be a drawback for their extrusion at room temperature. Besides, natural biopolymers, such as alginate and chitosan, which present inherent biocompatibility and high–water content, have to be specifically processed for printable purposes.^26^

In this work, the needed requirements to identify a suitable additive for creating tailored 3D nanoporous HAp and for the subsequent polarized catalysts have been focused on the rheological properties, which must be adequate for precisely controlling the final architecture and porosity of the samples without affecting their crystal structure. Therefore, the polymeric additive must be removed by the sintering process without affecting the dimensional and mechanical stability of the final HAp scaffold. In a recent study, Hodásová et al. reported the utilization of Pluronic F-127 hydrogel for creating 3D-printed highly porous yttrium-stabilized zirconia scaffolds, fulfilling all the conditions aforementioned.^29^ Therefore, as a proof of concept, we explore the use of pluronic hydrogels intended to control the porosity and rheological properties of HAp inks for the fabrication of highly nanoporous polarized catalysts. Here, we report, for the first time, the possibility of creating nanoporous HAp scaffolds at low temperatures by using Pluronic F-127 hydrogel. More specifically, scanning electron microscopy (SEM), Raman microscopy, wide-angle X-ray diffraction (WAXD), and nanoindentation have been used to elucidate the structure, including nanopore generation, and mechanical properties of HAp scaffolds prepared by using different hydrogel concentrations. Finally, nanoporous HAp scaffolds have been catalytically activated by applying the TSP treatment. The performance of both the conventional and nanoporous catalysts has been compared for different carbon and nitrogen fixation reactions, the latter showing an improved catalytic performance that boosts the reaction yields.

## Methods

### Synthesis of Nanoporous HAp Inks

HAp powder, hereafter denoted as prepared HAp, was obtained by the hydrothermal route and freeze-dried for 72 h to eliminate the water content. The hydrogel was prepared mixing Pluronic F-127 with water. Details about the preparation of HAp and the Pluronic F-127 hydrogel are provided in the Supporting Information.

HAp inks with desired weight percentage of Pluronic F-127 hydrogel were obtained from the slow addition of half of the weighted between the hydrogel to HAp powder, followed by rigorous stirring at 2500 rpm for 2 min using a Fisherbrand digital vortex mixer. This process was repeated again adding the rest of hydrogel to achieve the homogeneous mixture. All the procedure was performed at low temperature (i.e., in a cold room at 4 °C). The obtained white paste (HAp ink) was left aging at 4 °C for 24 h to ensure that the Pluronic F-127 hydrogel became homogeneously distributed. Finally, HAp inks were modeled at low temperatures to obtain the desired 3D HAp scaffolds and sintered at 1000 °C by using a muffle Carbolite ELF11/6B/301 for 2 h. Hereafter, this product is denoted as s/*x*-HAp, where s refers to sintered and *x* corresponds to the mass percentage (%) of pluronic hydrogel. In terms of completion, sintered grains based on as prepared HAp powder were also prepared by using the same temperature and time conditions (s-HAp).

### Characterization

Structural characterization was performed by using wide-angle X-ray diffraction (WAXD), Raman microscopy, scanning electron microscopy (SEM), and energy dispersive X-ray (EDX) analyses. Water absorption capabilities were obtained by means of contact angle measuring equipment. Details are provided in the Supporting Information.

The mechanical properties at the nanometric length scale, mainly hardness (*H*) and elastic modulus (*E*), were measured by using the nanoindentation technique. Tests were performed with a Nanoindenter XP (MTS) unit, equipped with a continuous stiffness measurement mode (CSM), allowing a dynamic determination of the mechanical properties during the indentation process.^30^ Indentations were arranged in a homogeneous spaced array of 400 imprints (20 × 20) conducted under loading control mode at 40 mN until reaching the maximum displacement into surface of 2000 nm. The distance between imprints was kept constant at 50 μm to avoid any overlapping effect. The strain rate was held constant at 0.05 s^–1^, and the indenter shape was carefully calibrated for true indentation depth as small as 25 nm by indenting fused silica standard of well-known Young’s modulus of 72 GPa.^31^ The values of *H* and *E* were directly determined by means of the Oliver and Pharr method^30,31^ and subsequently assessed by employing the statistical methodology proposed by Ulm and Constantinides.^32−35^ More information related to this protocol is available in the literature.^36,37^

### Catalytic Activation

s-HAp disks, which were obtained by pressing 150 mg of s-HAp powder at 620 MPa for 10 min, and s/*x*-HAp cubes were catalytically activated by placing the samples between two stainless steel plates (AISI 304) and applying a constant DC voltage of 500 V for 1 h with a GAMMA power supply, while temperature was kept at 1000 °C. Samples were allowed to cool, maintaining the applied electric potential for 30 min, and finally, all the system was powered off and left to cool overnight. Hereafter, catalysts derived from s-HAp and s/*x*-HAp have been denoted as HAp/c and *x*-HAp/c.

### Carbon and Dinitrogen Fixation Reactions

Activated catalysts were introduced in an inert reactor and tested for three reported electrothermal catalytic reactions: (1) the synthesis of simple amino acids from N_2_, CO_2_, and CH_4_;^38^ (2) the synthesis of ethanol from CO_2_ and CH_4_;^9^ (3) the synthesis of NH_3_ from N_2_. Quantification of the reaction yields was performed by using ^1^H NMR spectroscopy. Specific details are provided in the Supporting Information.

## Results and Discussion

**Scheme 1:**
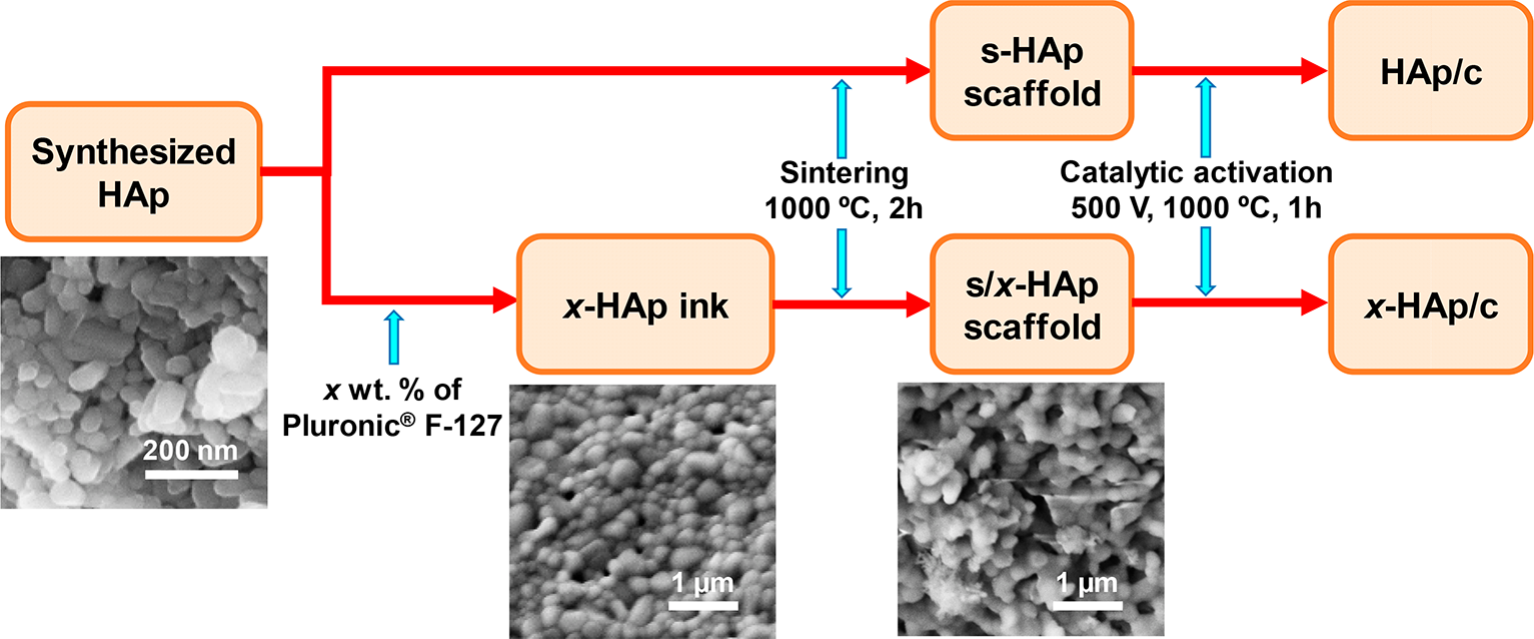
Processes Used to Prepare HAp/c and *x*-HAp/c

In this work, the structure and efficiency of conventional (i.e., poorly porous) and nanoporous HAp catalysts have been compared. The conventional catalyst (HAp/c) was prepared by polarizing discs obtained pressing sintered HAp pellets (s-HAp), as described in previous work.^9,38^ Porous and nanoporous catalysts were prepared by polarizing the sintered scaffolds obtained by using mixtures of HAp pellets and Pluronic F-127 hydrogel, which have been denoted s/*x*-HAp (where *x* corresponds to the mass percentage of hydrogel). The porous and nanoporous catalysts derived from s/*x*-HAp have been denoted *x*-HAp/c. Accordingly, s-HAp and HAp/c have been compared with s/*x*-HAp and *x*-HAp/c, respectively. The processes used to prepare HAp/c and *x*-HAp/c are summarized in Scheme 1.

### Structural Characterization

To achieve optimal catalytic activation, as-prepared HAp powder was calcined at 1000 °C, enhancing the crystallinity, surface charge accumulation, and OH^–^ vacancies through a dehydration process.^11^ Moreover, exposure of HAp inks at such high temperature is also necessary to confer mechanical stability and to eliminate Pluronic F-127 hydrogel, which could interfere in the catalyst performance (i.e., masking the catalytic activity). In this section, the structure and crystallinity of s-HAp and s/*x*-HAp are compared with the initial as-prepared HAp.

**Figure 1:**
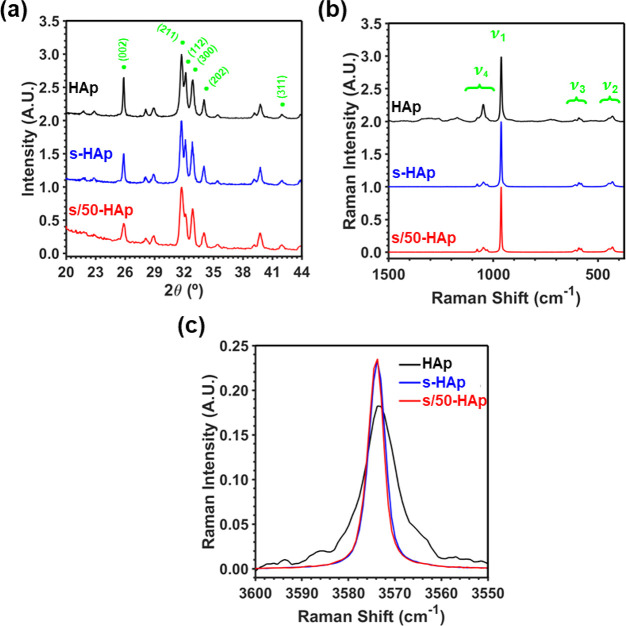
Structural characterization of as-prepared HAp, s-HAp, and s/50-HAp samples: (a) WAXD spectra, (b) Raman spectra in the region of the four characteristic vibrational modes (ν_1_–ν_4_), and (c) stacked Raman spectra of the samples in the region of 3550–3600 cm^–1^ corresponding to the ν(O–H) vibrational mode.

WAXD spectra of as-prepared HAp, s-HAp, and s/50-HAp samples are displayed in Figure 1a. All samples presented the characteristic peaks of HAp at 2θ = 25.9°, 31.7°, 32.1°, 32.8°, 34.0°, and 39.8°, which correspond to the (002), (211), (112), (300), (202), and (310) reflections, respectively (JCPDS card number 9-0077). Representative crystallographic parameters (Table 1) were obtained to determine whether the suppression of Pluronic F-127 hydrogel affected the dehydration process. The expected crystal refinement was observed for the two samples treated at high temperatures, slightly increasing their crystallinity (χ_c_; eq S1) from 0.78 ± 0.01 for HAp to 0.84 ± 0.02 for s/50-HAp and 0.81 ± 0.02 for s-HAp. However, some structural differences arose when the crystallite size of the (211) main reflection (*L*_211_) was analyzed (eq S2). A reduction of ∼4 nm was observed for s/50-HAp, suggesting that the presence of the hydrogel restricted the crystal growth during the crystal refinement process. This observation and the higher crystallinity of s/50-HAp indicate that the generation of pores and nanopores promotes the formation of crystallization nuclei. Therefore, crystals are more abundant but slightly smaller for s/50-HAp than for s-HAp, increasing the total catalytic active surface of the former.

**Table 1:** Representative Crystallographic Parameters Obtained for As-Prepared HAp, s-HAp, and s/50-HAp Samples

	Hap	s-HAp	s/50-HAp
χ_c_	0.78 ± 0.01	0.81 ± 0.02	0.84 ± 0.02
*L*_221_ [nm]	24 ± 2	24 ± 1	20 ± 1
*L*_002_ [nm]	54 ± 3	39 ± 2	24 ± 2
*I*_002_	0.585 ± 0.02	0.438 ± 0.02	0.238 ± 0.02
*I*_112_	0.728 ± 0.04	0.717 ± 0.03	0.598 ± 0.05

This observation was supported by the intensities of the (002) and (112) reflections normalized by the intensity of the (211) main reflection (*I*_002_ and *I*_112_, respectively), which were considerably lower for s/50-HAp (Table 1). *I*_002_ is commonly used to study the crystal growth anisotropy through the *c*-axis of the HAp lattice. The *I*_002_ values listed in Table 1 reflect the effect of the sintering of HAp grains (*I*_002_^HAp^ > *I*_002_^s-HAp^ > *I*_002_^s/50-HAp^). These results allow to conclude that the hydrogel acts as a template, directing the shape and size of HAp grains. This results in the generation of higher porosity.

Generation of other calcium phosphate phases, such as β-tricalcium phosphate (β-TCP), can be easily produced during the dehydration process. Although the characteristic (021) reflection of β-TCP, which appears at 2θ = 31.5° (JCPDS card number 9-0432), is not observed in the WAXD spectra, the formation of other salts was not completely discarded as most of their reflections share peak positions with HAp. In addition, Raman microscopy measurements were performed to obtain more detailed information about the phase distribution of the sintered samples.

Raman spectra, which are shown in Figure 1b, present the characteristic vibrational fingerprint of HAp (corresponding to the PO_4_^3–^ internal modes), enabling to discard both the Pluronic F-127 hydrogel residues (since no peaks related to the employed hydrogel identity were detected) and the presence of other calcium phosphate phases. Thus, the latter would be unequivocally identified by a characteristic splitting or a shifting of the main peak at ν_1_*=* 962 cm^–1^, which corresponds to the P–O symmetric stretching mode. Moreover, such peaks become sharper and better defined after sintering, indicating that crystallinity increases. Additionally, the areas of the peak at 3574 cm^–1^, which are associated with the O–H stretching mode, were evaluated and normalized by the area of the ν_1_ (*A*_3574_) to control the proper generation of OH^–^ vacancies during the dehydration process (Figure 1c).^11^ Results clearly show that the presence of Pluronic F-127 hydrogel does not affect the final amount of generated vacancies, as long as *A*_3574_*=* 0.160 ± 0.003 and 0.167 ± 0.002 for s-HAp and s/50-HAp are almost 33% times lower than the value obtained for as-prepared HAp. Therefore, the sintering process successfully removes the hydrogel from the HAp scaffold without affecting the crystalline structure of the mineral.

### Influence of Pluronic F-127 Hydrogel on the Final HAp Scaffolds

**Figure 2:**
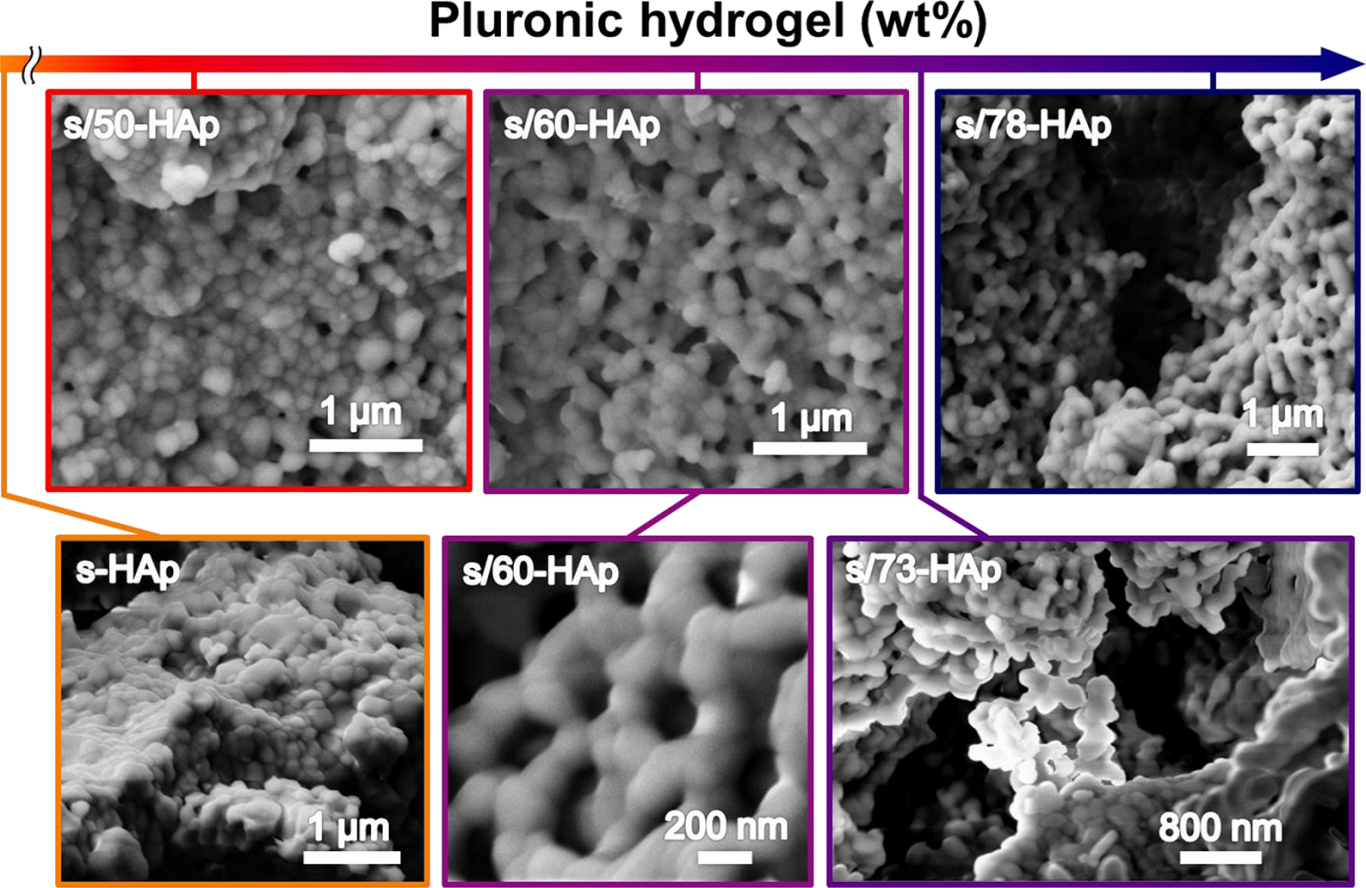
SEM micrographs of s-HAp and s/*x*-HAp samples obtained using different Pluronic F-127 hydrogel mass percentages. Porosity is clearly observed when the hydrogel was introduced into the blend.

Figure 2 compares SEM micrographs of s-HAp and s/*x*-HAp, which were obtained by using different Pluronic F-127 hydrogel mass percentages (i.e., 50, 60, 73, and 78 wt %). The generation of spherical nano- and submicrometric pores (from 120 ± 73 to 400 ± 122 nm) was clearly observed in all s/*x*-HAp samples. Nano- and submicrometric pores consistently present normal distributions for all samples (Figure S1), even though a new family of micrometric cavities appear when the mass percentage of the employed hydrogel increases from 50 to 73 and 78 wt % (Figure 2). Such a phenomenon has been attributed to the effect of residual hydrogel that was not properly dispersed among the HAp powder. Furthermore, the sizes of micrometric pores present a poor dependence on the wt % of Pluronic F-127 hydrogel (6.2 ± 2.0 and 5.4 ± 2.8 μm for s/73-HAp and s/78-HAp, respectively), coexisting both types of pores independently. The micrometric cavities induced by the higher percentage of hydrogel should be associated with more fragile samples, as is reported in the literature.^39^

**Figure 3:**
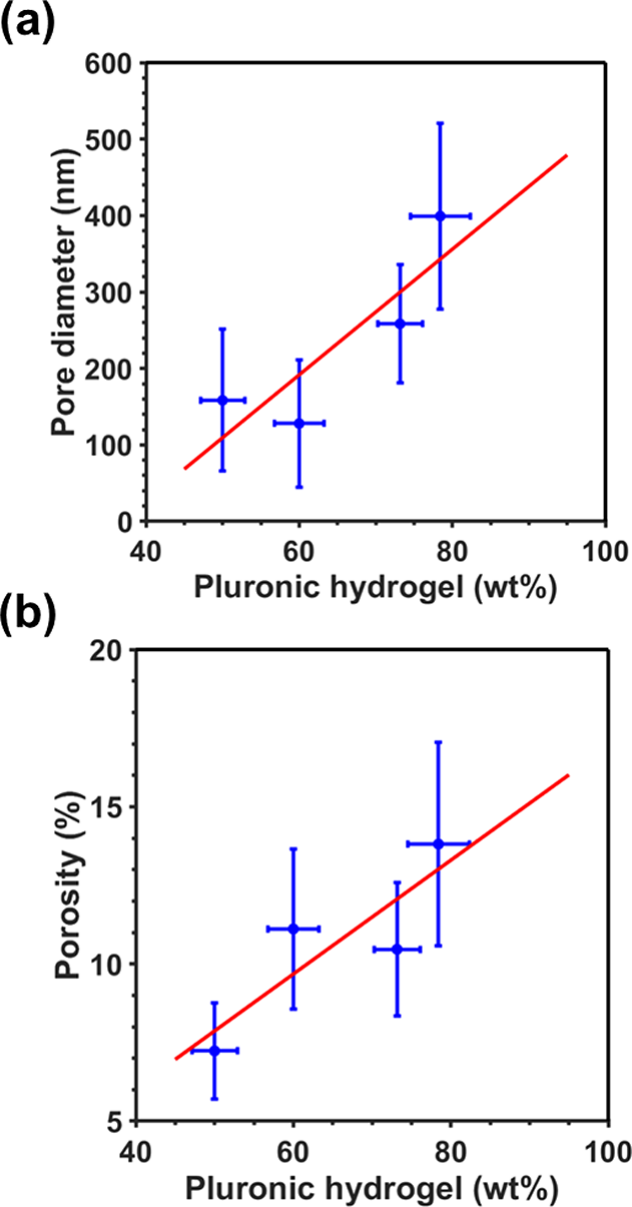
Dependence of (a) the pore size and (b) the porosity of s/*x*-HAp samples on the Pluronic F-127 hydrogel wt %.

The porosity of samples associated with nano- and submicrometric pores (i.e., discarding micrometric cavities), which was obtained considering the percentage of void space observed in SEM micrographs, ranges from 6 to 14%.^40^ High-magnification SEM micrographs (Figure 2) indicate that Pluronic F-127 hydrogel droplets are the precursors of the resulting pores, favoring the location of HAp sintered grains in their surroundings. Figure 3 shows that the pore size and the porosity of s/*x*-HAp depend on the size and number of hydrogel droplets, evidencing that the former can be tailored by controlling the mass percentage of hydrogel added to the initial mixture.

**Figure 4:**
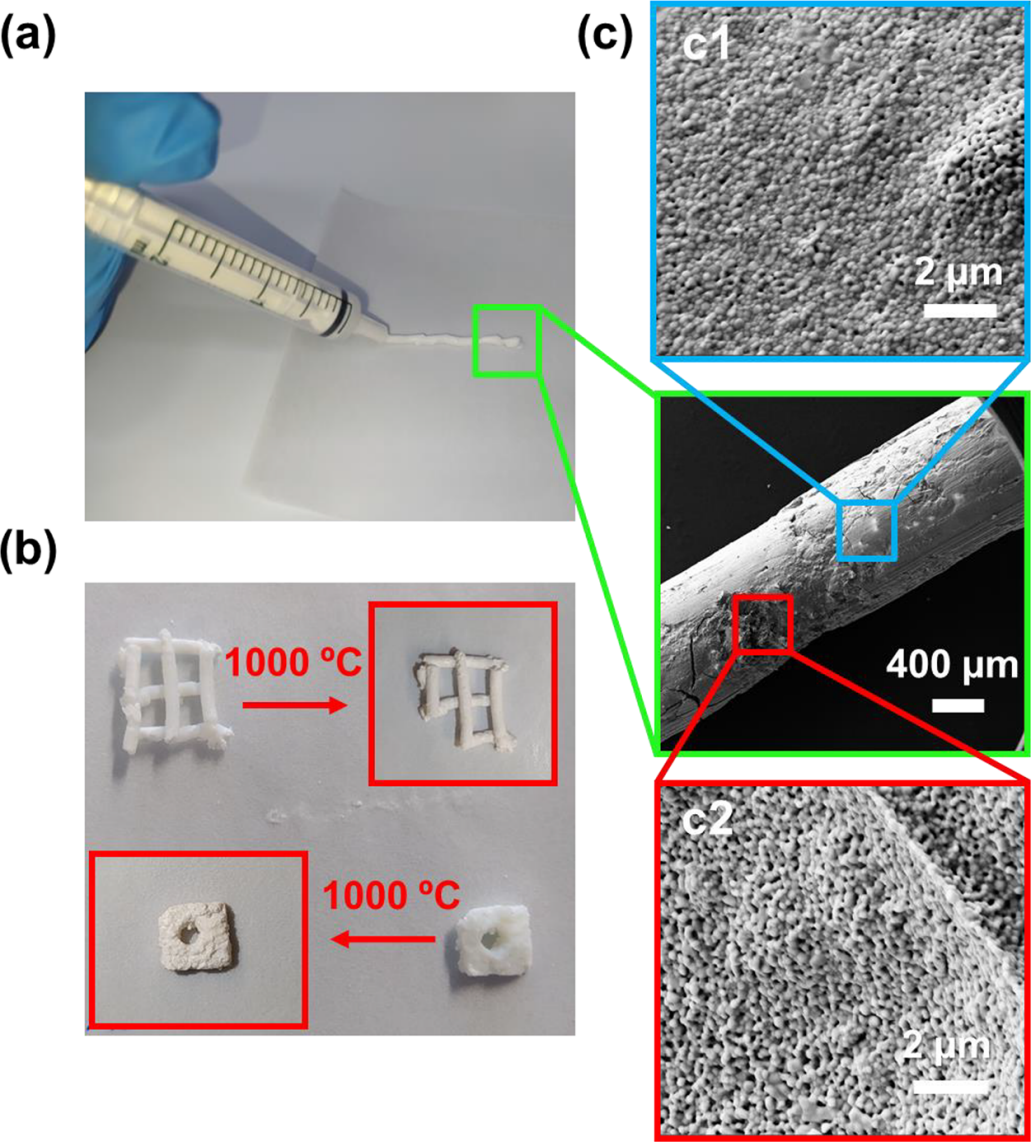
(a) HAp ink loaded with a 60 wt % of Pluronic F-127 hydrogel extruded with a syringe. (b) Effect of sintering on the structure of the same HAp ink, as modeled with different shapes. (c) SEM inspection of porosity the outer face (c1) and the interior (c2) of a HAp ink.

3D-printed HAp scaffolds were achieved by extruding the Pluronic F-127 hydrogel–HAp mixture, which exhibits good rheological conditions when maintained at low temperatures (<4 °C), as the hydrogel binds the HAp powder. To obtain the optimum paste properties for 3D printing, different HAp inks were preliminary examined by varying the mass percentage of the hydrogel (Figure S2). Although the catalytic efficiency is expected to increase with the exposed catalytic surface area and, therefore, with the content of Pluronic F-127 used in the ink, other properties should be also considered to choose the most advantageous content of hydrogel in the ink. These are the ease of shaping the ink and the mechanical integrity of the scaffolds. As is discussed below, two such properties are much more advantageous for the systems prepared by using a hydrogel content of 60 wt % than for those derived from a hydrogel content of 78 wt %. Accordingly, a Pluronic F-127 hydrogel mass percentage of ∼60 wt % was considered for further studies due to its optimal printable properties and its good balance between the mechanical stability and both the control of pore size and the porosity. Figure 4a,b shows that such HAp ink is viscous enough to be introduced into a syringe but sufficiently dry to maintain the desired shape even at cold temperatures, when Pluronic F-127 hydrogel is still liquid. Moreover, sintered samples maintain the desired shape without showing observable cracks or shrinkage and with good structural stability with fair bonding between filaments, as shown in Figure 4b.

SEM micrographs acquired from extruded filaments revealed that the porosity at the exposed surface decreased to 4% (Figure 4c1), which has been attributed to the friction of the HAp ink with the walls, suggesting that such behavior could be a drawback. However, focusing on a broken region of the rod where the inner part (or the cavity) can be observed, the porosity recovers the standard value of 12% (Figure 4c2), which is in agreement with that found for s/60-HAp samples (Figure 3b). Even though the surface porosity could be enhanced by using coated syringes and/or more viscous HAp inks, together with a precise control of the temperature, our results indicate that the utilization of a syringe presents significant handling advantages.

Microporosity and nanoporosity, which have been exploited in the biomedical field to enhance cell adhesion and diffusion of proteins due to capillary forces,^14^ are expected to play a decisive role in the reactions catalyzed by polarized HAp. Similarly, proper wettability is also desired for the heterogeneous catalytic fixation of carbon and/or nitrogen from molecular gases due to (1) improved gas diffusion through the active sites of the catalyst and (2) the presence of water in the solid–liquid–gas catalyst interface as it has been determined to be one of the reaction limiting factors.^9^ Accordingly, water absorption capability studies were conducted to compare s-HAp and s/60-HAp scaffolds (Figure S3). To enhance their porosity, s/60-HAp scaffolds were shaped as cubes (Figure 4b), whereas s-HAp consisted on disks made of compressed HAp powder. Table S1 shows that water flow absorption was almost 4 times greater for s/60-HAp than for s-HAp, which was mainly attributed to the pores of the former.

The same study was performed after catalytic activation of s-HAp and s/60-HAp, which produced the HAp/c and 60-HAp/c catalysts by applying the TSP process. Identical experimental conditions (Methods section) were applied to both samples. Although the polarization process is known to affect the hydrophilicity of the samples due to a surface charge induction effect,^12^ the enhancement of water absorption capability was only observed for HAp/c, which was attributed to the non-negligible effect of increased roughness. Despite this, 60-HAp/c still presents a much better absorption capability than HAp/c (i.e., 1.5 times greater), which is expected to promote the final catalytic activity of the carbon and nitrogen fixation reactions.

**Figure 5:**
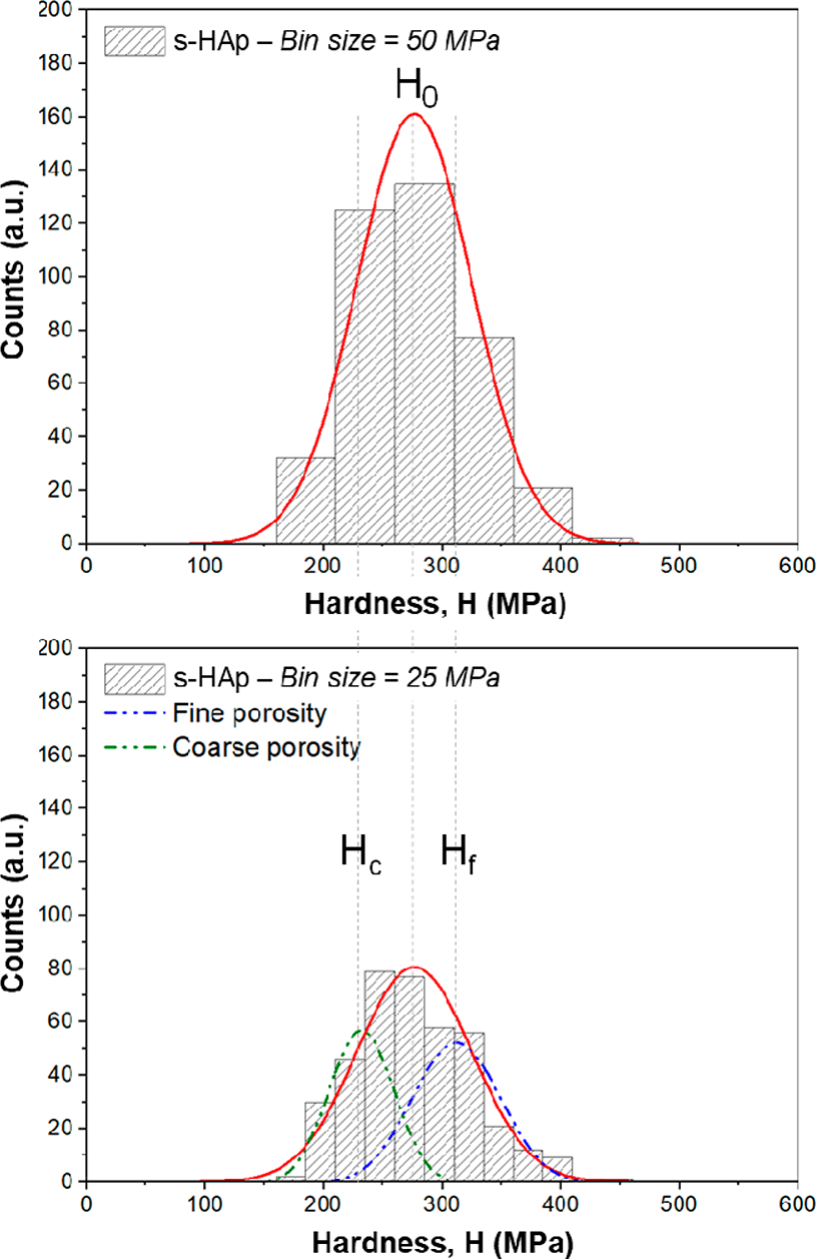
Histogram of hardness values (bin size 50 and 25 MPa) determined from 400 indents performed with a 40 mN load. A substructure is observed, dependent on the histogram bin size.

The mechanical behavior of s-HAp, s/60-HAp, and s/78-HAp specimens was studied at the micro- and submicrometric length scale by means of the nanoindentation technique under loading control mode. Figure 5 displays the histogram of the measured *H* for s-HAp with a constant bin size of 50 and 25 MPa, obtained from an average of 400 imprints. The histogram with a constant bin size of 50 MPa presents a monomodal pore size distribution, while that obtained by using a bin size of 25 MPa exhibits two different peaks. The first peak corresponds to the imprints where the plastic (for hardness measurement) and elastic (for elastic modulus determination) flows are affected by relatively coarse porosity, and the second one corresponds to the imprints affected by a fine porosity (labeled as peaks 1 and 2, respectively, in Table 3) heterogeneously distributed inside the specimen. Qualitatively similar histograms were obtained for the other specimens and are not shown here.

**Table 3:** Summary of the Hardness (*H*) and Elastic Modulus (*E*) Values of Each Pore Distribution Interaction and for Each Specimen Determined from Statistical Analysis^a^

	peak 1		peak 2	
specimen	*H* (GPa)	*E* (GPa)	*H* (GPa)	*E* (GPa)
s-HAp	232.0 ± 7.5	10.2 ± 0.2	311.1 ± 6.8	21.5 ± 0.3
s/60-HAp	179.9 ± 5.2	8.2 ± 0.2	280.3 ± 2.5	19.5 ± 0.1
s/78-HAp	138.2 ± 5.8	7.9 ± 0.1	b	
s/60-HAp “hole-shaped”	50.3 ± 4.3	6.5 ± 0.3	114.5 ± 5.2	15.4 ± 0.2

a The bin size was held constant and is equal to 25 MPa for both investigated properties.

b This specimen only presents one peak distribution.

The values obtained by using a constant bin size of 25 MPa for each peak (for the coarse and fine pores and labeled with a subindex *c* and *f* in Figure 5) presented above and for each specimen are summarized in Table 3. The *H* and *E* values were around 25% and 15% lower for porous samples than for s-HAp, respectively. Such reductions increased to around 70% and 30%, respectively, for hole-shaped porous specimens shaped with hole in the center (see Figure 4b).

### Catalytic Activity of 60-HAp/c

It is worth noting that the catalytic properties of polarized HAp were attributed to both the generation of vacancies and the polarization of OH^–^ groups.^11^ Thus, the superficial charge, which exhibited a linear dependence with number of vacancies, was demonstrated to be directly related to permanently polarized HAp catalytic properties.^11^ Moreover, the permanent polarization of OH^–^ groups (i.e., dipole pointing to specific direction) allowed electric charges to move freely across different crystalline domains, resulting in the “electrical leakages” necessary for the reactions. Permanently polarized HAp was also investigated by studying its resistive and capacitive behavior by using electrochemical impedance spectroscopy (EIS).^41^ Such results showed that the thermally stimulated polarization treatment delocalizes the charge carriers of the bulk over crystal domains, accumulating at the surface of the samples. These phenomena drastically decrease the resistance at the interface and reduce the capacitive imperfections due to grain boundaries, leading to an ideal capacitive behavior and providing electrocatalytic properties. The effect of this electric mechanism is expected to be drastically enhanced by the increment of the catalytic surface, which is significantly higher in 60-HAp/c than HAp/c due to the pores induced by the mixture with Pluronic F-127 hydrogel.

To elucidate the effect of porosity on the catalytic activity of the HAp-based catalysts, 60-HAp/c cubes were prepared and compared with HAp/c disks (Scheme S1). For this purpose, s/60-HAp cubes and s-HAp disks were polarized by using identical experimental conditions, which are described in the Methods section. The porosity was calculated in previous sections considering SEM micrographs of grains and comparing different Pluronic F-127 hydrogel charges. However, the utilization of specific geometrical parameters, as defined in Scheme S1, instead of grains allows an alternative expression of relative porosity (Π_rel_) measured by gravimetry, which appears to be more appropriate for comparing both samples from a catalytic point of view. Hence
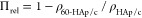
1where ρ_60-HAp/c_ and ρ_HAp/c_ are the densities of 60-HAp/c cubes and HAp/c disks, respectively, which were obtained by using the parameters described in Table S2. The resulting value, Π_rel_*=* 0.7 ± 0.03, indicates that the difference between the porosities of the materials is higher than the one obtained by SEM inspection. Indeed, Π_rel_ suggests greater porosity with good pore interconnectivity in the bulk and confirms the fact that external handling of the HAp inks results in a reduction of superficial porosity due to abrasion.

The catalytic performances of the 60-HAp/c and HAp/c catalysts were compared for three different reactions based on nitrogen and carbon fixation: (i) the synthesis of amino acids using CO_2_, CH_4_, and N_2_ mixtures;^38,42^ (ii) the production of ethanol using mixtures of CO_2_ and CH_4_;^9^ and (iii) the conversion of N_2_ to ammonia. As is shown below, the performance of 60-HAp/c as catalyst largely depends on the requirements of the reaction. Thus, both the porosity and water absorption capability of 60-HAp/c represent serious drawbacks for the production of amino acids, the yield decreasing several orders of magnitude with respect to HAp/c. On the contrary, the conversion of CO_2_ and CH_4_ to ethanol and of N_2_ to ammonia improves by around 3000 and 2000%, respectively, in comparison to HAp/c, as discussed below. On the other hand, it should be mentioned that reaction mechanisms as well as the corresponding blanks were described in previous works and therefore have not been discussed in this work, which is exclusively focused on the comparison between the catalytic performances of 60-HAp/p and HAp/c.

#### Synthesis of Amino Acids Using CO_2_, CH_4_, and N_2_

The synthesis of glycine (Gly) and alanine (Ala) was conducted under a N_2_, CO_2_, and CH_4_ atmosphere irradiated with UV light (more details can be found in the Supporting Information), as reported in previous work.^38,42^ To obtain those amino acids from carbon and nitrogen fixation, it is necessary to coat the HAp-based catalyst by incorporating two layers of aminotris(methylenephosphonic acid) (ATMP) separated by one layer of zirconyl chloride (ZC). Layers were prepared by depositing 100 μL of the corresponding ATMP or ZC solutions onto 60-HAp/c cubes or HAp/c discs. After deposition of each coating layer, samples were dried at room temperature for at least 8 h before deposition of the next layer. Although the roles of ATMP and ZC layers were exhaustively discussed in previous works,^8,38^ it is worth noting that the catalytic active site is based on the three components at the interface (i.e., HAp/c-ATMP/ZC/ATMP), which is controlled by the concentration of ATMP and ZC solutions used to incorporate the different layers.^38^ However, the porosity and the enhancement of water absorption capability observed for 60-HAp/c may be relevant factors for influencing the final morphology of the coating.

To study the formation of each layer on the 60-HAp/c surface, SEM micrographs were recorded for the catalyst coated with ATMP and ZC layers obtained by using solutions with different concentrations (Figure S4 and Table S3). For HAp/c, ATMP nucleated forming separate islands that expanded in width and height, covering and masking the HAp/c surface as the concentration increases.^38^ On the contrary, the porosity of 60-HAp/c promotes the wettability and, therefore, the creation of much thinner but larger ATMP coating layers, even at low concentrations. Interestingly, the presence of ATMP flowerlike structures found for HAp/c when applying the third ATMP layer^38^ was not observed for 60-HAp/c samples. Instead, the apparition of large cracks was detected (Figure S4), which has been attributed to the increased water absorption capability.

**Figure 6:**
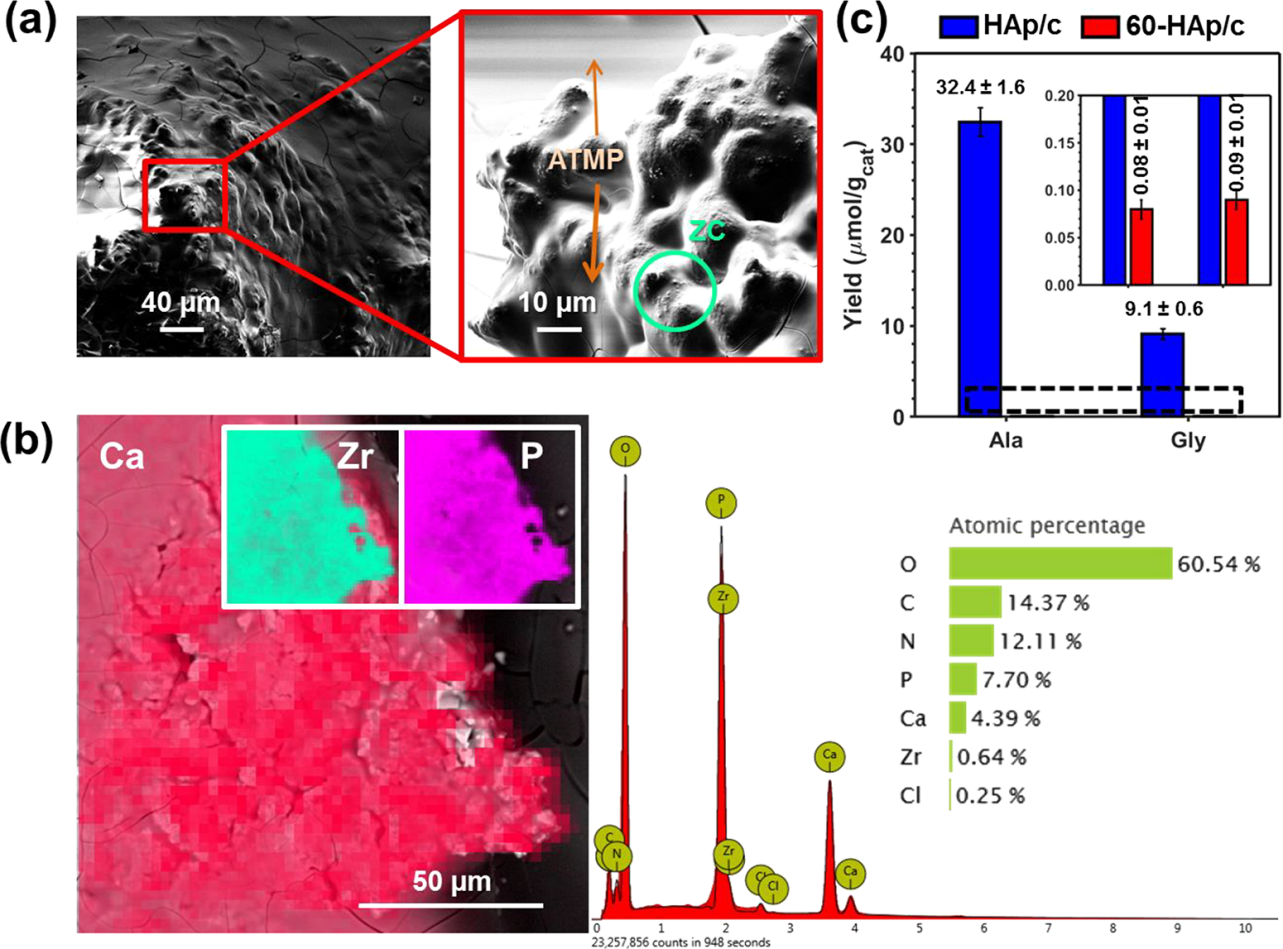
(a) SEM micrograph of the 60-HAp/c catalyst coated with an ATMP/ZC/ATMP 3-layer, which was prepared by using 5 mM ATMP and ZC solutions. ATMP and ZC have been highlighted. (b) EDX results for the sample displayed in (a). (c) Yields of Ala and Gly were obtained by using p-HAp/c and HAp/c coated with ATMC/ZC/ATMP. The yields were derived from ^1^H NMR measurements. The reaction conditions were the following: CO_2_, CH_4_, and N_2_, 2 bar each; 95 °C under UV light for 48 h.

Figure 6a shows a representative SEM micrograph of the 60-HAp/c catalyst coated with three layers (ATMP/ZC/ATMP), which were prepared by using 5 mM solutions of ATMP and ZC. EDX analyses were used to confirm the presence and distribution of both ATMP and ZC (Figure 6b). Despite such low concentration, the surface is homogeneously coated by the third ATMP layer, hindering the surface pores of 60-HAp/c. Moreover, EDX confirmed the existence of a ZC homogeneous layer in between, stressing out the differences found in other HAp/c-coated systems.^38^ As such hindering is even more pronounced in catalysts coated by using more concentrated ATMP and ZC solutions, the one displayed in Figure 6a,b was chosen for the reaction involving the production of Gly and Ala.

Figure 6c compares the Gly and Ala yields of the reactions catalyzed by HAp/c and 60-HAp/c ATMP/ZC/ATMP-coated catalysts. The yield of both Gly and Ala is much lower for 60-HAp/c (0.09 ± 0.01 and 0.08 ± 0.01 μmol per gram of catalyst for Gly and Ala, respectively) than for HAp/c (9.1 ± 0.6 and 32.4 ± 1.6 μmol per gram of catalyst for Gly and Ala, respectively). This feature confirms that the exposed ATMP-ZC-HAp interface is lower for 60-HAp/c than for HAp/c. Overall, no enhancement of catalytic activity was observed for this reaction. On the contrary, the amino acids final yields were drastically reduced, which was attributed to the improved wettability of the 60-HAp/c. This property favors the creation of homogeneous ATMP and ZC layers, hindering the pores and blocking the diffusion of gases through the microcavities of the catalyst and blocking the transfer of active species from the catalyst to the active sites.

#### Production of Ethanol Using CO_2_ and CH_4_

**Figure 7:**
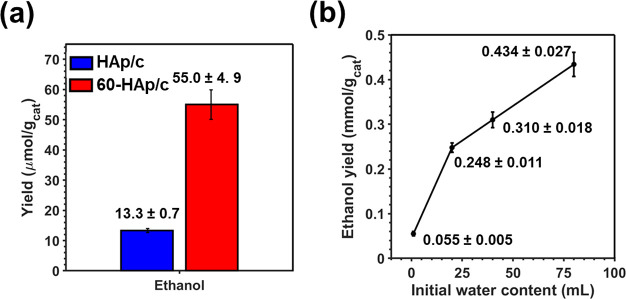
(a) Comparison of the ethanol yields obtained from the CO_2_ and CH_4_ fixation reaction as catalyzed by HAp/c and 60-HAp/c. Reactions were performed by using an initial CO_2_:CH_4_ mixture (3 bar each), 1 mL of water, at 140 °C without UV irradiation for 48 h. (b) Variation of the ethanol production as a function of the initial water content (expressed in mmol/g_cat_ instead of μmol/g_cat_). The rest of the reaction conditions were identical with those described for (a).

The limitations associated with the utilization of 60-HAp/c for the synthesis of amino acids were not expected for the production of ethanol, as the latter process does not require the coating of the catalyst. In recent studies, we reported the selective synthesis of ethanol by carbon fixation from CO_2_ and CH_4_ gas mixtures using naked HAp/c as catalyst.^9^ For this work, the performances of 60-HAp/c and HAp/c catalysts for the production of ethanol were compared by loading a CO_2_:CH_4_ mixture (3 bar each) and 1 mL of liquid water into the reaction and applying 140 °C for 48 h without UV irradiation. The ethanol yields obtained for both 60-HAp/c and HAp/c are compared in Figure 7a. The ethanol production was around 4 times higher for the former than for the latter (55.0 ± 4.9 and 13.3 ± 0.7 μmol per gram of catalyst, respectively), which represents an outstanding increment of the catalytic performance (414%).

Previous studies evidenced that water is necessary as proton source, whereas the excess of water content hinders the gas fixation onto the HAp/c surface, diminishing the final yields of the reaction.^9^ In this work we examined if the water content was still a limiting factor for 60-HAp/c, which is characterized by both the generation of pores and the high water absorption in comparison to HAp/c. Accordingly, a series of reactions were conducted varying the initial water content from 1 to 80 mL, whereas the CO_2_ and CH_4_ pressures (3 bar each), the temperature (140 °C), and the reaction time (48 h) were kept.

The yield of ethanol, which is plotted in Figure 7b, increased progressively with the amount of water initially introduced in the reactor, even when the initial water content was 80 times greater than of the used in the standard reaction for HAp/c (1 mL). This result describes the huge catalytic potential of 60-HAp/c that can produce up to 434 ± 27 μmol per gram of catalyst when the water content is 80 mL, reaching for the first time to a production comparable to the one reported for cutting edge Cu-based catalysts.^43^ Moreover, it is worth noting that Cu-based catalysts require the application of electric potentials,^43^ whereas no potential is necessary for 60-HAp/c performance. On the other hand, ethanol or any other product in the remaining water inside the reactor (referred as supernatant in previous work^9^) was found to be null, highlighting the absorption capability of the catalyst and stressing out the scalability of it. Overall, it can be concluded that porosity boosts the catalytic performance of HAp/c for the production of ethanol, obtaining for the first time yields high enough for industrial scalability and economic feasibility.

#### Conversion of N_2_ to Ammonia

The production of NH_4_^+^ using 60-HAp/c and HAp/c catalysts was performed at 120 °C by using under UV illumination. To eliminate the initial air content, the reaction chamber was first purged with N_2_ and, subsequently, filled with N_2_ until reach a pressure of 6 bar. A volume of 20 mL of deionized water was introduced in the reactor and put in contact with the nonirradiated side of the catalyst. It is worth noting that in this reaction water acts not only as the proton source for the ammonia formation (from water splitting) but also as a medium to facilitate the recovery of the formed product. The product generated on the surface of the catalysts as well as the product collected in liquid water after 24 h of reaction was identified as NH_4_^+^ adapting a procedure based on ^1^H NMR spectroscopy.^44^

Results, which are depicted in Figure S5, showed that the total amount of NH_4_^+^ formed in the presence of HAp/c was of 6.2 ± 0.9 μmol per gram of catalyst. Although around 25% of the formed NH_4_^+^ (i.e., 1.7 ± 0.3 μmol per gram of catalyst) remained adsorbed onto the catalyst surface, the main part of the reaction product was transferred from the catalyst to the water medium (i.e., 4.5 ± 0.6 μmol per gram of catalyst). As it was expected, the influence of the porosity and water absorption capacity on the yield of NH_4_^+^ was dramatic, the amount of product formed in the presence of 60-HAp/c increasing to 128.8 ± 22.3 μmol per gram of catalyst. Moreover, the total of the yield, which represented an increment of more than 2000% with respect to HAp/c, was collected on the remaining liquid water. This feature, which is fully consistent with the results obtained in the previous reaction, corroborates that 60-HAp/c enhances the synthesis of the reaction products.

## Conclusions

Nanoporous HAp scaffolds with tailored architecture have been successfully created by mixing HAp powder with Pluronic F-127 hydrogel. This composition of this mixture allows to regulate the properties of the resulting ink to fulfill 3D-printing requirements, the proper mechanical stability being achieved by sintering at high temperatures. Sintered HAp scaffolds exhibit high purity and crystallinity, reflecting a correct dehydration process. Therefore, the addition of Pluronic F-127 hydrogel for enabling the printable inks, and their posterior generation of nanopores, does not affect the structure required for preparing polarized HAp-based catalysts.

The catalytic activity of the porous samples has been evaluated by using different carbon and/or dinitrogen fixation reactions. Although the utilization of coatings hinders the effect of pores, the naked 60-HAp/c catalyst shows an outstanding increment of the yields of the reaction. This has been mainly attributed to the enhanced water absorption capability and higher exposed surface. More specifically, the presence of microcavities inside the catalyst promotes the heterogeneous catalytic processes used for the production ethanol and ammonia. Overall, its catalytic activity and huge scalability potential postulate 60-HAp/c as a solid, cheaper, and environmentally more friendly alternative to other conventional catalysts.

## Supporting Information Available

Experimental methods, geometry and size of the samples, water absorption capacity, pore size distribution diagrams, photographs of the inks, SEM micrographs, and ammonium yields (PDF)
