# Supplementary material for: Tailorable Nanoporous Hydroxyapatite Scaffolds for Electrothermal Catalysis
Source: ACS Appl Nano Mater. 2022 May 20;5(6):8526–36. doi: 10.1021/acsanm.2c01915 (PMC9989946; doi:10.1021/acsanm.2c01915)
Supplement: Supplementary file 1 — an2c01915_si_001.pdf [file an2c01915_si_001.pdf]

SUPPORTING INFORMATION

Tailorable Nanoporous Hydroxyapatite Scaffolds for Electrothermal Catalysis

Jordi Sans,[1,2,] Marc Arnau,[1,2] Joan Josep Roa[2,3] Pau Turon[4,*] and Carlos Alemán[1,2,5,*]*

[1] *Departament d'Enginyeria Química, EEBE, Universitat Politècnica de Catalunya, C/ Eduard Maristany, 10-14, Ed. I2, 08019, Barcelona, Spain*

[2] *Barcelona Research Center in Multiscale Science and Engineering, Universitat Politècnica de Catalunya, C/ Eduard Maristany, 10-14, 08019, Barcelona, Spain*

[3] *CIEFMA-Departament de Ciència i Eng. de Materials, Universitat Politècnica de Catalunya, Eduard Maristany 10-14, Ed. I, 08019 Barcelona, Spain*

[4] *B. Braun Surgical, S.A. Carretera de Terrasa 121, 08191 Rubí (Barcelona), Spain*

[5] *Institute for Bioengineering of Catalonia (IBEC), The Barcelona Institute of Science and Technology, Baldiri Reixac 10-12, 08028 Barcelona Spain*

[*] Correspondence to: jordi.sans.mila@upc.edu, pau.turon@bbraun.com and carlos.aleman@upc.edu

Page	Description
S2-S6	Experimental methods.
S6	Scheme S1. Geometrical comparison of 60-HAp/c cubes and HAp/c disks.
S7	Table S1. Water absorption capability.<br>Table S2. Macroscopic parameters of the 60-HAp/c and HAp/c catalysts.<br>Table S3. Coating conditions for the synthesis of amino acids.
S8	Figure S1. Pore size distribution diagrams.
S8	Figure S2. Photographs corresponding to different discarded HAp inks.
S9	Figure S3. Representative water absorption capability photographs.
S10	Figure S4. SEM micrographs corresponding to the samples listed in Table S1.
S11	Figure S5. Comparison of the ammonium yields obtained from the N_2 fixation reaction as catalyzed by HAp/c and 60-HAp/c.

METHODS

Materials

Calcium nitrate $Ca(NO_3)_2$, diammomium hydrogen phosphate [$(NH_4)_2HPO_4$; purity > 99.0%], ammonium hydroxide solution 30% [NH_4OH; purity: 28-30% w/w], zirconyl chloride ($ZrOCl_2 \cdot 8H_2O$; ZC), aminotris(methylenephosphonic acid) (ATMP) and the initial Pluronic® F-127 polymer ($(C_3H_6O \cdot C_2H_4O)_x$, BioReagent powder) were purchased from Sigma Aldrich. Ethanol (purity > 99.5%) was purchased from Scharlab. N_2, CH_4 and CO_2 gases with a purity of > 99.995% were purchased from Messer. All experiments were performed with milli–Q water.

Synthesis of Hydroxyapatite (HAp)

15 mL of 0.5 M $(NH_4)_2HPO_4$ in de-ionized water were added at a rate of 2 mL/min to 25 mL of a 0.5 M of $Ca(NO_3)_2$ solution in ethanol with pH previously adjusted to 11 using ammonium hydroxide solution. The mixture was left aging for 1 h under gentle agitation (150 rpm) at room temperature. Hydrothermal treatment at 150 ºC was applied using an autoclave Digestec DAB–2 for 24 h. The autoclave was allowed to cool down before opening. The precipitates were separated by centrifugation and washed with water and a 60/40 v/v mixture of ethanol/water (twice). After freeze–drying it for 3 days, a white powder was obtained.

Synthesis of Pluronic® F-127 hydrogel

25 g of distilled water was mixed with 25 g of Pluronic® F-127 polymer using a FlackTek SpeedMixer at 3500 rpm for 5 minutes. After that, 50 g of Pluronic® polymer were added and vigorously stirred using the same conditions. The resultant hydrogel was stored at 4 ºC.

Characterization

Structural analyses were performed by means of an inVia Qontor confocal Raman microscope (Renishaw) equipped with a Renishaw Centrus 2957T2 detector and a 532 nm laser. In order to obtain representative data, 32 single point spectra were averaged.

Wide angle X-ray diffraction (WAXD) studies were conducted using a Brucker D8 Advance model with Bragg-Brentano 2θ configuration and Cu K_α radiation ($\lambda = 0.1542$ nm). Measurements were performed in a 2θ range of 20º–60º in steps of 0.02º and scan speed of 2 s, using a one-dimensional Lynx Eye detector. The crystallinity (χ_c) was obtained using the following expression:

$$\chi_c = 1 - \frac{V_{112/300}}{I_{300}} \quad \text{(S1)}$$

where I_{300} is the intensity of the (300) reflection and $V_{112/300}$ is the intensity of the hollow between the (112) and (300) reflections, which disappears in non-crystalline samples. The crystallite size, L_{hkl}, was calculated using the Debye-Scherrer equation

$$L_{\mathrm{hkl}} = \frac{0.9 \cdot \lambda}{B \cdot \cos\theta_{\mathrm{hkl}}} \quad \text{(S2)}$$

where λ is the wavelength of the monochromatic X-ray beam, B is the full width at half maximum of the peak at the maximum intensity, and θ_{hkl} is the peak diffraction angle that satisfies the Bragg's law for the (hkl) plane.

SEM studies were carried out using a Focused Ion Beam Zeiss Neon40 microscope operating at 5 kV equipped with an EDX (20 kV) spectroscopy system. The latter technique was used to estimate the composition of the distribution of the ATMP/ZC/ATMP coatings.

Water absorption capability was computed by means of a contact angle measuring equipment OCA 15EC (Data-Physics Instruments). To dispense the water droplet, a 500 μL DS500/GT glass syringe and a needle SNS 021/011 were used. The absorption flow rates were calculated by dropping 1.5 μL water droplets onto the surface of the samples while recording with a 30 fps camera. Then, the photograms were analyzed.

Synthesis of Amino Acids

The catalytic synthesis of glycine (Gly) and alanine (Ala) by means of carbon and dinitrogen fixation were prepared using HAp/c or 60-HAp/c catalysts, which were coated with two layers of ATMP separated by an intermediate layer of ZC (*i.e.* ATMP/ZC/ATMP). ATMP and ZC layers were prepared by depositing 100 μL of the corresponding solutions. Different concentrations of each coating solution, which are listed in Table S1, were tested. After deposition of each coating solution, samples were dried at room temperature for at least 8 h before the deposition of the next layer.

The reactor consisted in an inert reaction chamber coated with a perfluorinated polymer (120 mL), in which both the catalyst and water (1 mL) were incorporated. The reactor was equipped with an inlet valve for the entrance of N_2, CH_4, CO_2 and an outlet valve to recover the gaseous reaction products. A UV lamp (GPH265T5L/4, 253.7 nm) was also placed in the middle of the reactor to irradiate the catalyst directly, the lamp being protected by a UV transparent quartz tube. All surfaces were coated with a thin film of a perfluorinated polymer

in order to avoid any contact between the reaction medium and the reactor surfaces, in this way discarding other catalyst effects.

Reactions were performed at 95 ºC for a reaction time of 48 h. Both HAp/c disks and 60-HAp/c cubes coated with ATMP/ZC/ATMP and 1 mL of de-ionized liquid water were initially incorporated into the reaction chamber (reactions were performed separately for each catalyst). The chamber was extensively purged with the first selected gas (N_2) in order to eliminate the initial air content. Each selected gas was introduced to increase the reaction chamber pressure (measured at room temperature) to the target pressure. In all cases the chamber pressure was increased up to 6 bar by introducing sequentially 2 bar of each feeding reaction gas (*i.e.* N_2, CH_4 and CO_2).

Synthesis of ethanol

The reaction was performed using the same reactor chamber under a CO_2 and CH_4 atmosphere (3 bar each), at 140 ºC but without the presence of UV light for 48 h. Initially, CO_2 was used to purge the reactor. The catalyst, HAp/c disks or 60-HAp/c cubes without any coating, and 1 mL of de-ionized liquid water were incorporated into the reaction chamber (reactions were performed separately for each catalyst). Additionally, the effect of the initial water content on the reaction yield was investigated for one of the 60-HAp/c catalysts.

Quantitative analyses

The reaction products were analyzed by ^{1}H-NMR spectroscopy. All ^{1}H-NMR spectra were acquired with a Bruker Avance III-400 spectrometer operating at 400.1 MHz. The chemical shift was calibrated using tetramethylsilane as internal standard. Sixty-four scans were recorded in all cases. In order to remove the products formed on the catalysts from reactions

involving CO_2 and CH_4, samples were dissolved in deuterated water containing 100 mM of HCl and 50 mM of NaCl with the final addition of deuterated water.

In the case of the dinitrogen fixation reaction to produce ammonia, the catalyst (10 mg) was dissolved in 15 mL of water with pH adjusted to 2.1 ± 0.2 using 7.6 mM H_2SO_4, to promote the conversion of ammonia in NH_4^+, and applying 4 cycles that involved sonication (5 min) and stirring (1 min) steps. Then, for the ^{1}H-NMR sample preparation, 500 µL of the reacted catalyst solution were mixed with 100 µL of DMSO-d_6 instead of solvents with labile deuterons (*i.e.* D_2O) to avoid the formation of ammonium deuterated analogues, not desired for quantitative analysis. The same treatment was applied to the water supernatant.

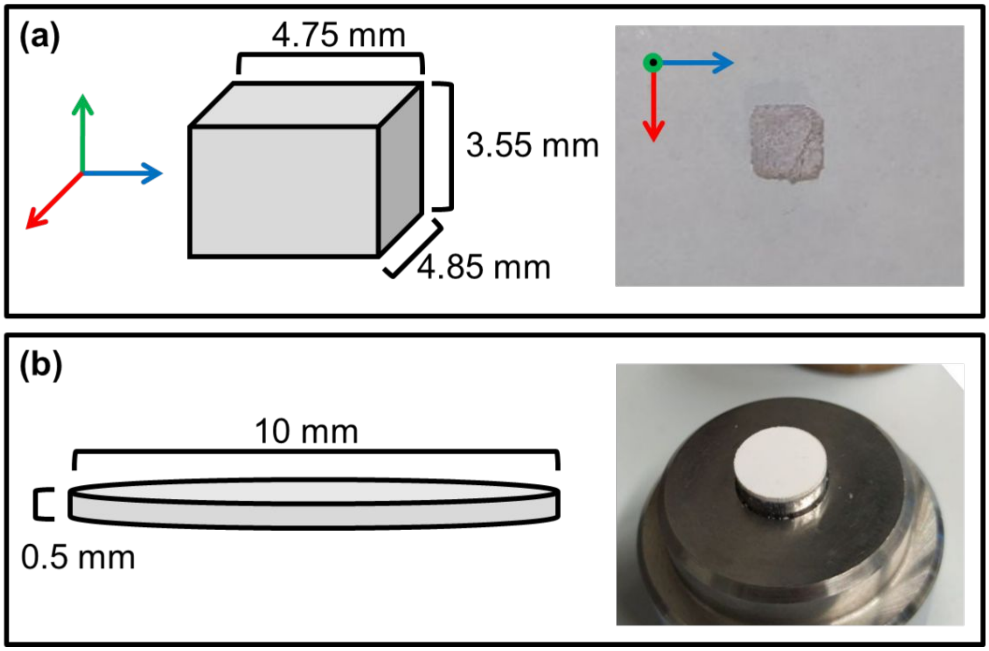

Scheme 2. Geometrical comparison of (a) 60-HAp/c cubes and (b) HAp/c disks.

Table S1. Water absorption capability of different samples before (s-HAp and s/60-HAp) and after (HAp/c and 60-HAp/c) catalytic activation.

	**Samples**			
	**s-HAp**	**s/60-HAp**	**HAp/c**	**60-HAp/c**
**Water flow absorption [µL/s]**	0.93 ± 0.14	3.57 ± 0.17	1.73 ± 0.39	2.60 ± 0.04

Table S2. Macroscopic parameters of the 60-HAp/c and HAp/c catalysts (Scheme S1). The exposed area only considers the faces of the geometry that are exposed in the reaction.

	**Exposed Area [mm^2]**	**Volume [mm^3]**	**Weight [mg]**
60-HAp/c (cube)	74.3	81.8	132
c-HAp (disk)	94.3	39.3	200

Table S3. Summary of the 60-HAp/c catalyst coated with ATMC and ZC layers obtained using solutions with concentrations. Single- bi- and three-layered systems have been studied. The 60-HAp/c-ATM(5mM)/ZC(5mM)/ATMP(5mM) corresponds to the catalysts used for the synthesis of amino acids.

**60-HAp/c-coated samples**	**1st layer (ATMP)**	**2nd layer (ZC)**	**3rd layer (ATMP)**
60-HAp/c-ATMP(50mM)	50 mM	-	-
60-HAp/c-ATMP(10mM)	10 mM	-	-
60-HAp/c-ATMP(10mM)/ZC(10mM)	10 mM	10 mM	-
60-HAp/c-ATMP(10mM)/ZC(10mM)/ATMP(10mM)	10 mM	10 mM	10 mM
60-HAp/c-ATMP(5mM)/ZC(5mM)/ATMP(5mM)	5 mM	5 mM	5 mM

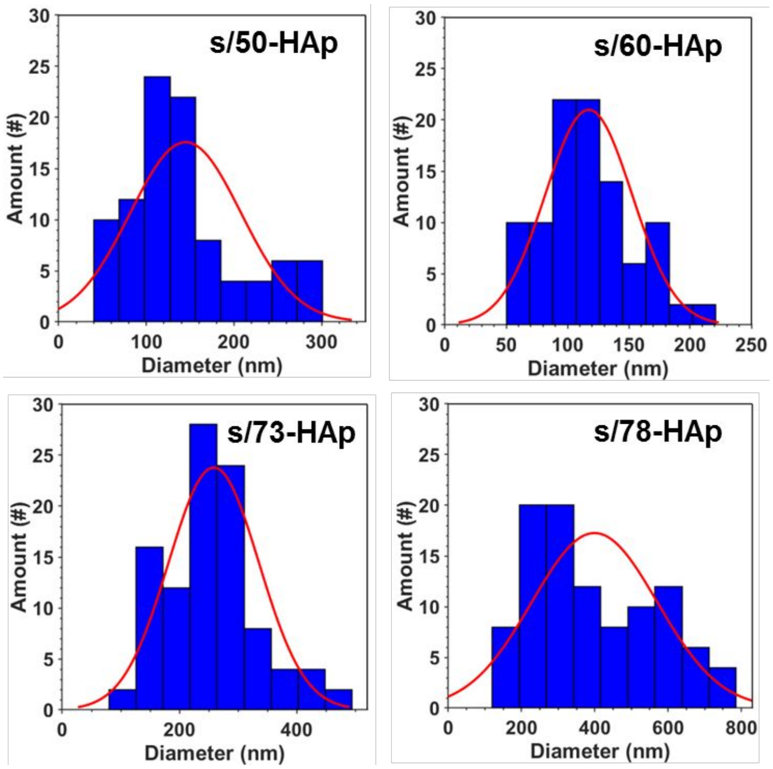

Figure S1. Pore size distribution histograms for s/*x*-HAp samples.

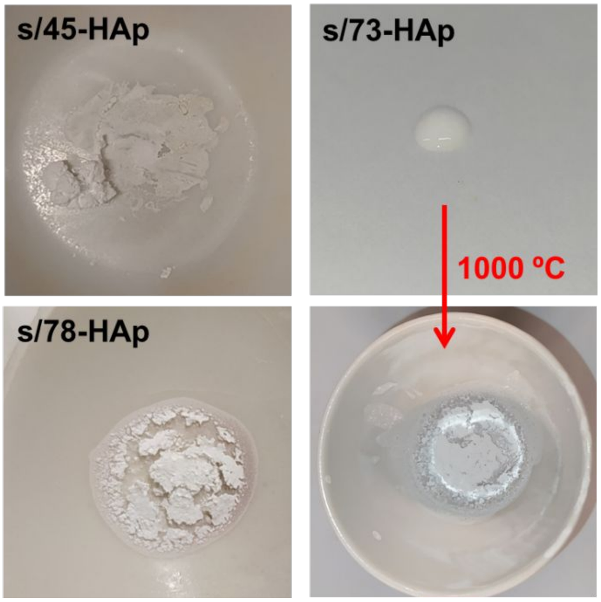

Figure S2. Determination of the optimum Pluronic® F-127 hydrogel (wt. %) loading in terms of printability and mechanical stability of the HAp inks.

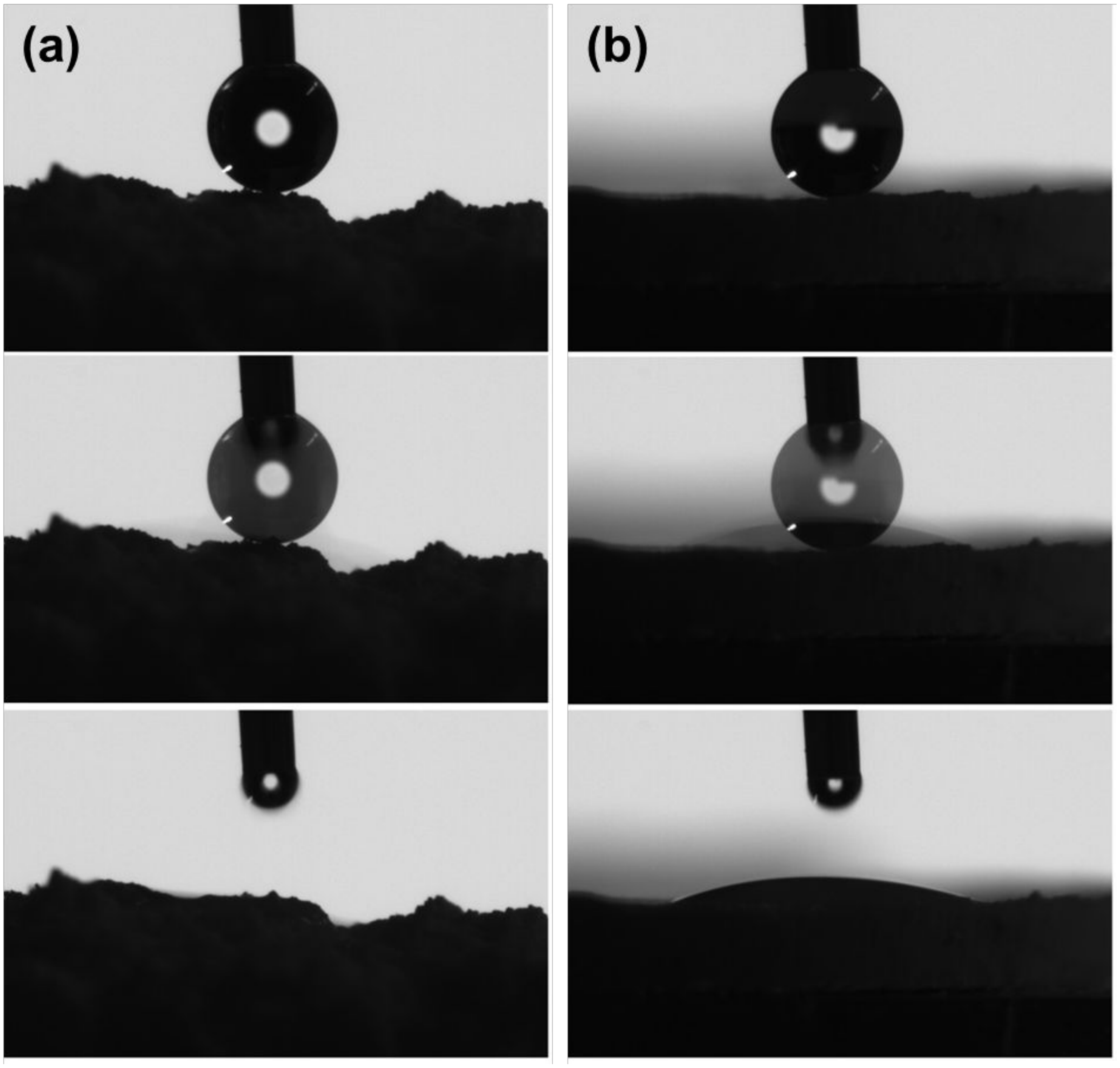

Figure S3. Representative photographs of the water absorption capability assays using contact angle tests for (a) s/60-HAp, and (b) s-HAp samples.

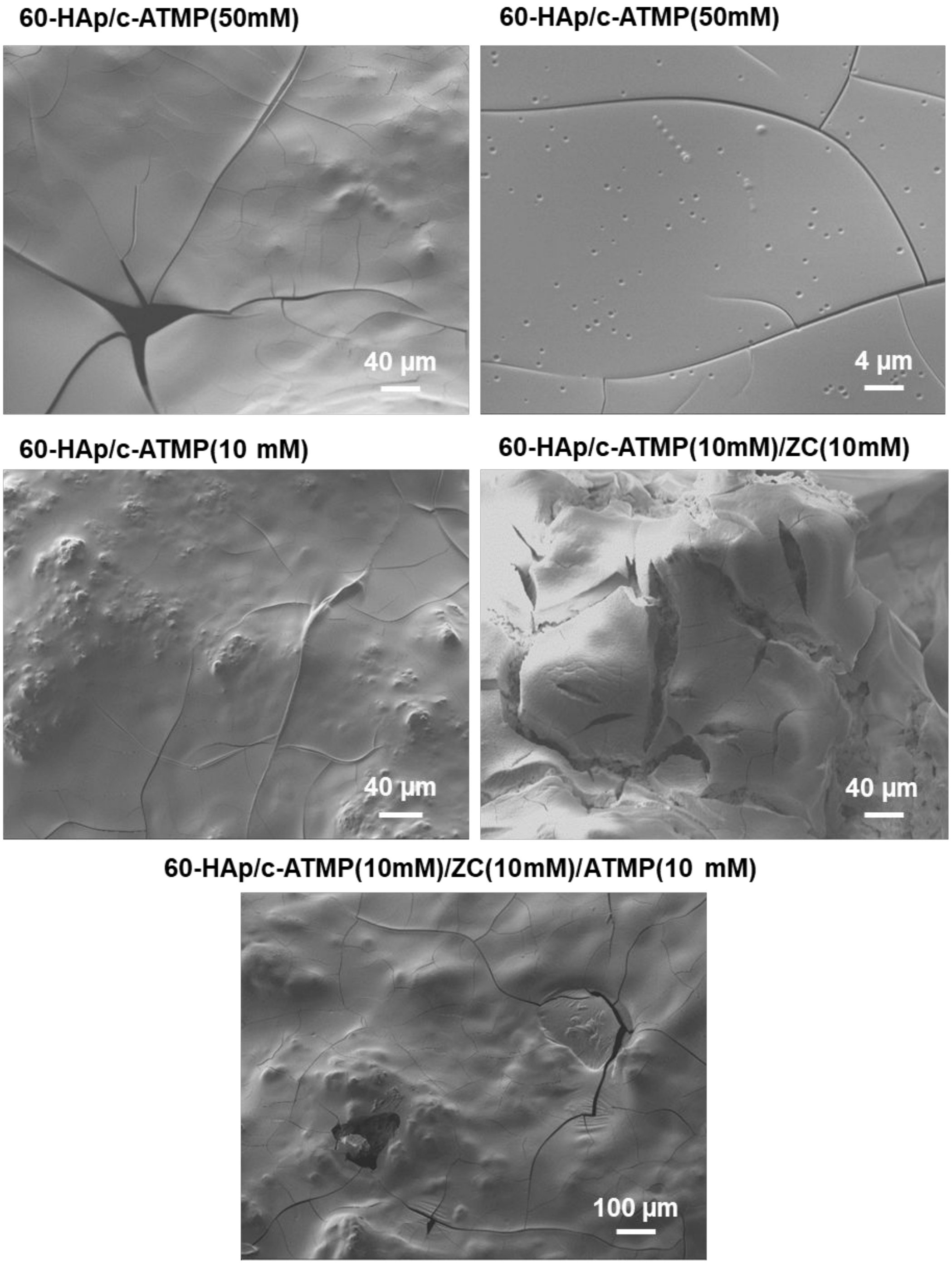

Figure S4. SEM micrographs of coated 60-HAp/c catalysts listed in Table S1. The nomenclature indicates the concentration (in mM) used for the different ATMP and ZC layers.

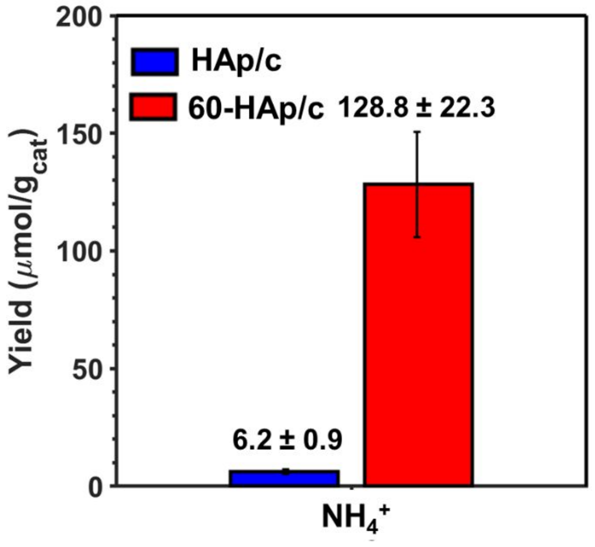

Figure S5. Comparison of the ammonium yields obtained from the N_2 fixation reaction as catalyzed by HAp/c and 60-HAp/c. Reactions were performed using an initial N_2 pressure (6 bar), 20 mL of water, at 120 ºC with UV irradiation for 24 h.
